# Unique N-glycosylation signatures in Aβ oligomer-and lipopolysaccharide-activated human iPSC-derived microglia

**DOI:** 10.21203/rs.3.rs-5308977/v1

**Published:** 2024-11-19

**Authors:** Xinyu Tang, Ryan Schindler, Jacopo Lucente, Armin Oloumi, Jennyfer Tena, Danielle Harvey, Carlito Lebrilla, Angela Zivkovic, Lee-Way Jin, Izumi Maezawa

**Affiliations:** University of California, Davis; University of California, Davis; University of California Davis Medical Center; University of California, Davis; University of California, Davis; University of California, Davis; University of California, Davis; University of California, Davis; University of California Davis Medical Center; University of California Davis Medical Center

**Keywords:** Microglia, amyloid, lipopolysaccharide, glycosylation, sialylation, fucosylation

## Abstract

Microglia are the immune cells in the central nervous system (CNS) and become pro-inflammatory/activated in Alzheimer’s disease (AD). Cell surface glycosylation plays an important role in immune cells; however, the N-glycosylation and glycosphingolipid (GSL) signatures of activated microglia are poorly understood. Here, we study comprehensive combined transcriptomic and glycomic profiles using human induced pluripotent stem cells-derived microglia (hiMG). Distinct changes in N-glycosylation patterns in amyloid-β oligomer (AβO) and LPS-treated hiMG were observed. In AβO-treated cells, the relative abundance of bisecting N-acetylglucosamine (GlcNAc) N-glycans decreased, corresponding with a downregulation of MGAT3. The sialylation of N-glycans increased in response to AβO, accompanied by an upregulation of genes involved in N-glycan sialylation (ST3GAL4 and 6). Unlike AβO-induced hiMG, LPS-induced hiMG exhibited a decreased abundance of complex-type N-glycans, aligned with downregulation of mannosidase genes (MAN1A1, MAN2A2, and MAN1C1) and upregulation of ER degradation related-mannosidases (EDEM1-3). Fucosylation increased in LPS-induced hiMG, aligned with upregulated fucosyltransferase 4 (FUT4) and downregulated alpha-L-fucosidase 1 (FUCA1) gene expression, while sialofucosylation decreased, aligned with upregulated neuraminidase 4 (NEU4). Inhibition of sialyation and fucosylation in AβO- and LPS-induced hiMG alleviated pro-inflammatory responses. However, the GSL profile did not exhibit significant changes in response to AβO or LPS activation. AβO- and LPS- specific glycosylation changes could contribute to impaired microglia function, highlighting glycosylation pathways as potential therapeutic targets for AD.

## INTRODUCTION

Neuroinflammation plays significant roles in the initiation and progression of several central nervous system (CNS) disorders, including Alzheimer’s disease (AD). The complexity of neuroinflammation reflects how microglia respond to internal and external changes to orchestrate inflammatory responses. Microglia are the innate immune cells of the CNS that survey and respond to various environmental challenges with multiple actions, resulting in phagocytosis of debris and release of cytokines and chemokines ^1^. Prior to the development of specific immunohistochemical agents, a widely used classic histochemical method to detect microglia in brain sections was to employ lectins that recognize glycoconjugates ^2^, implying the significance of glycosylation (i.e., post-translational addition of glycans to protein backbones) in microglial function. Glycosylation is tightly regulated due to its important role in the immune system. Alterations in glycans can significantly affect interactions between immune system components ^3^. For example, sialylated glycans likely function as self-associated molecular patterns, interacting with receptors like sialic acid-binding immunoglobulin-type lectins (Siglecs) to modulate immune responses ^4^. Furthermore, glycosylation changes have been observed in various cancers and autoimmune diseases, such as rheumatoid arthritis ^5^, ovarian cancer ^6^, gastric cancer ^7^, and lung cancer ^8^. Recently, the study of changes in glycosylation upon neuroinflammation is gaining increased interest ^9^; however, how microglia respond to stimuli with glycosylation changes remains understudied.

AD pathology has highlighted the importance of microglia given their role in orchestrating neuroinflammation and phagocytosis. In AD brains, microglia lose their homeostatic molecular signature and show profound functional impairments, such as increased production of pro-inflammatory cytokines, elevated reactive oxygen species, impaired phagocytosis, and increased inflammasome formation ^10^. Recently, the pivotal roles of microglia-orchestrated neuroinflammation in AD have been established ^11–14^. A generally accepted hypothesis states that activated microglia become functionally impaired and release cytotoxic substances and pro-inflammatory cytokines that cause neuronal damage and aggravate AD pathology ^14^. While multiple factors may cause microglial activation in AD, early studies have established that different species of amyloid-β (Aβ) aggregates are potent stimulants of microglia. Among them, we and others found that the small soluble Aβ oligomer (AβO) assembled from Aβ42 peptide provides far stronger stimulation to induce microglial activation ^15,16^. Aβ aggregates are recognized by a range of microglial pattern recognition receptors to induce mainly pro-inflammatory responses that could mediate Aβ-induced neurotoxicity, impair phagocytic function, and prime microglia to enhance their sensitivity and reactivity to inflammatory stimuli ^17^. Understanding mechanisms of such activations could reveal microglial therapeutic targets.

In the present study, we characterized the global glycosylation changes of human microglia following AβO stimulation, using stimulation with lipopolysaccharide (LPS), a widely used microglia activation approach, for comparison. We employed human induced pluripotent stem cell (iPSC)-derived microglia (hiMG) as a major model rather than the widely used primary cultures from neonatal rodents based on the following rationales. First, it was recognized that rodents are generally a better model for neuronal pathology than they are for microglial pathology ^18^, and that for optimal translational validity, human microglia are recommended to be used to identify human-relevant molecular pathways and therapeutic targets ^19^. Second, in contrast to the evolutionarily better conserved genetic codes and protein networks across species, there is no evidence for a universal “glycan code” akin to the genetic code or protein motif ^20,21^. Rather, glycans vary immensely in structure and expression both within and between evolutionary lineages, and our knowledge about the glycan structural diversity between species remains limited ^21^. Therefore, we consider that models of human cells would be suitable for our initial investigation on microglia glycosylation changes. Our profiling of hiMG identified the specific glycan types and glycosylation genes altered in AβO- and LPS-activated human microglia that may be involved in the regulation of their respective activation pathways.

## METHODS

### Human iPSC culture and microglia differentiation

Human iPSCs were obtained from ALSTEM.INC. (Richmond, CA). The line used in this study was Human iPS Cell Line 26 (Episomal, CD34+, ApoE3). Cells were plated onto Matrigel (Fisher) coated plates and cultured with mTeSR plus (Stemcell Technology). For microglia differentiation, we followed a previously described protocol ^22,23^. Briefly, 2 × 10^6^ iPSCs were plated onto Aggrewell 800 plates (Stemcell Technology) to form embryoid bodies (EBs) in mTeSR1 supplemented with Bone Morphogenetic Protein 4 (BMP4, 50 ng/ml)/ Vascular Endothelial Cell Growth Factor (VEGF, 50 ng/ml)/ Stem Cell Factor (SCF, 20 ng/ml) and culture for four days with daily medium change. On the fifth day, EBs were plated onto gelatin coated 6-well plates with 20 EBs per well in X-VIVO15 (Lonza) supplemented with M-CSF (100 ng/ml), IL-3 (25 ng/ml), Glutamax (2 mM), Penicillin/streptomycin (100 U/ml and 100 ug/ml) and β-mercaptoethanol (55 uM), and the medium was changed weekly. After 3–4 weeks, floating primitive macrophage precursor (PMP) were collected and plated onto 12-well plates (5 × 10^4^ cells/well), 6-well plates (3 × 10^5^ cells/well), or 100 mm dishes (1.5 × 10^6^ cells) and differentiated in microglia differentiation medium (Advanced DMEM/F12 supplemented with IL-34 (100 ng/ml), GM-CSF (10 ng/ml), N2 supplement (1x), Glutamax (2 mM), Penicillin/streptomycin (100 U/ml and 100 ug/ml) and β-mercaptoethanol (55 uM)) for two weeks. The cultures were routinely tested for mycoplasma.

For AβO and LPS stimulation, cells were treated with AβO (3 μM) and LPS (100 ng/ml) for 24 hrs. Each group, including the Control group, has six replicates.

### AβO preparation

AβO composed of Aβ1-42 peptide was prepared following a standard procedure ^24^ with a modification that the HFIP treated Aβ1-42 peptide (Bachem) was dissolved in DMSO and then diluted with Advanced DMEM/F12 culture medium instead of the F12 medium originally described, followed by incubation at 4°C for 24 hr and 10 min centrifugation at 10,000 ×rpm at 4°C. This preparation of AβO has been extensively characterized in our laboratory ^16^. Briefly, to ensure consistency of quality, a random sample from each batch was chosen and imaged using electron microscopy and atomic force microscopy to characterize the size and shape of the aggregates. The biological activities of each batch were confirmed by determining for AβO the neurotoxic activity, synaptic binding activity, and ability to rapidly induce exocytosis of MTT formazan, as described previously ^16^.

### RNA isolation, processing, and sequencing

Total RNA from cultured cells was extracted using RNeasy Plus Mini Kit (Qiagen). RNA quality evaluation (yield, purity and integrity), cDNA library construction and Illumina sequencing were performed by NovoGene (Sacramento, CA, USA) using 2 × 150 bp paired-end run. A total of 24 samples, comprising four groups (AβO vs Control and LPS vs Control) with six replicates per group, were sequenced with the output of 53.3±8.2 M reads per sample.

### Mapping and differential expression analysis

Adapter sequences in the raw FASTQ files were trimmed using Trimmomatic (v0.39). The remaining reads were mapped to the human reference genome hg38 using HISAT2 (v2.2.1) ^25^. The mapping rate was around 97%. The gene counts were summarized by featureCounts (v2.0.1) ^26^, achieving a successful alignment of 70%−80% of the reads per library. Differential gene expression was analyzed using edgeR package ^27^ in R version 4.1.0 (R Foundation for Statistical Computing, Vienna, Austria). Low-expressed genes were dropped using the *filterByExpr()* function. The filter step keeps genes that have count-per-million (CPM) above 10 in 70% of samples. The library sizes were recalculated after filtering. Sample- specific effects were removed by normalizing library sizes with the trimmed mean of M-values (TMM) method using the *calcNormFactors()* function. A negative binomial model with the quasi-likelihood (QL) F-test was applied to perform the differential expression analysis among diagnosis groups. First, gene counts with normalized library sizes were fitted to a negative binomial generalized linear model. Then, the dispersion was estimated using the *estimateDisp()* function. The QL dispersion estimation was calculated using the *glmQLFit()* function, followed by the *glmQLFTest()* function that conducted the quasi-likelihood (QL) F-test. Finally, we used the *topTags()* function to output significantly differentially expressed genes. P-values were adjusted for multiple hypothesis testing using Benjamini & Hochberg (BH) with a threshold of adjusted p-value ≤ 0.05.

### Sialylation and fucosylation inhibition

For the application of glycosylation inhibitors, microglia were pre-incubated with 100 μM 2FF (2-Fluorofucose, SynChem Inc) or 100 μM 3FS (2,4,7,8,9-Penta-O-acetyl-N-acetyl-3-fluoro-D-neuraminic acid methyl ester, Carbosynth) for 1 hr before the addition of AβO (3μM) or LPS (100ng/ml). Twenty-four hrs later, cells were lysed for the following experiments.

### qPCR

Total RNA from cultured cells was extracted using RNeasy Plus Mini Kit (Qiagen). cDNA was synthesized using 100 ng RNA and iScript Reverse Transcription Supermix (BioRad). Quantitative real-time polymerase chain reaction (qPCR) was performed using SsoFast^™^ EvaGreen Supermix and CFX96 qPCR system (BioRad). The forward/reverse primer sequences used are listed in Table 1. Gene expression was normalized to an endogenous gene, β-actin. Relative cDNA levels for the target genes were analyzed by the 2-ΔΔCt method.

### Cell membrane enrichment

Samples were prepared for glycomic analysis based upon previously published methodologies ^28^. Briefly, harvested cells were centrifuged at 2,000 RCF for ten minutes after which the cell media was exchanged with 1.2 mL of 0.25 M sucrose (MilliporeSigma, Cat# S7903), 20mM HEPES (Thermo Fisher Scientific, 1 M, Cat# 15630080) adjusted to pH 7.4 with KOH (MilliporeSigma, Cat# P5958) and a 1:100 protease inhibitor cocktail (Calbio-chem, Cat# 539137) in water and pipette mixed gently. Cells were then lysed by sonication where a total of 60 joules of energy were applied to each sample over a one-minute period. The homogenate was centrifuged at 2,000 RCF for 10 minutes at 4°C and then the supernatant was transferred to 1.5 mL ultracentrifuge tubes and the nucleus precipitate was discarded. The cell membrane was precipitated by ultracentrifugation at 42,000 RCF for 45 minutes at 4°C after which the cytoplasmic supernatant was discarded taking care not to disturb the membrane pellet. Samples were resuspended in 0.5 mL of 0.2 M Na2CO3 (MilliporeSigma, Cat# P5958) in water and ultracentrifuged again after which the supernatant, containing membrane-associated proteins, was discarded. A final ultracentrifugation step using the same conditions with 0.5 mL of water was done to wash and desalt the samples, discarding the supernatant after.

### N-glycan sample preparation

The enriched membrane fraction was then resuspended in 200 μL of 100 mM NH4HCO3 (MilliporeSigma, Cat# 09830), 5 mM Dithiothreitol (Promega, Cat# V3151) in water to assist in protein denaturing. After gentle homogenization by pipette mixing, samples were placed in a 100°C water bath for two minutes with 10 second heating and cooling intervals to further denature the membrane proteins. After samples cooled to room temperature, 2 μL of PNGase F (New England Biolabs, Cat #P0705L) was added to enzymatically hydrolyze the N-glycans from their protein substrates. Enzymatic cleavage and conversion of the resulting reducing end amides to hydroxyls occurred at 37°C for 18 hours. Sample were brought to a total volume of 600 μL with water and then ultracentrifuged using the previously described parameters. The supernatant containing the released N-glycans was transferred to a new vial and the remaining membrane pellet was stored at −20°C until ready for glycosphingolipid processing. N-glycan samples were cleaned up using a porous graphitic carbon solid phase extraction plate (PGC-SPE, Glygen, Cat# FNSCAR800). SPE wells were first washed with elution solvent consisting of 80% acetonitrile, 0.1% trifluoracetic acid in water (v/v) and then conditioned in wash solvent consisting of 40% acetonitrile, 0.05% trifluoracetic acid in water (v/v). Samples were loaded onto the solid phase and washed three times with 600 μL of washing solvent and all liquids were discarded. N-glycans were then eluted with 600 μL of elution solvent into a new collection plate. For each SPE step, the PGC plate was centrifuged at 150 RCF to quickly ensure solvent flow-through. Enriched N-glycan samples were concentrated by drying to completion in vacuum centrifugation and reconstituted in 30 μL of water prior to analysis.

### N-glycan analysis methodology

Released N-glycan analysis was conducted with an Agilent 1200 series ChipCube system operating in positive ion mode and a 6520 Q-TOF mass spectrometer. Chromatographic separations were carried out utilizing a PGC-Chip II, 40 nL enrichment column, 75 × 43 mm analytical column, 5 μm (Agilent Technologies, G4240-62003). Mobile phase A (MPA) composition was 3% acetonitrile and 0.1% formic acid in water (v/v). Mobile phase B (MPB) composition was 90% acetonitrile, and 1% formic acid in water (v/v). Samples were cooled with a 4°C sample tray and 5μL was injected by the autosampler, loaded onto the enrichment column, and washed for 10 minutes with 100% MPA at 3 μL/min. Next, the chip valve switched to analysis mode moving the enrichment column in line and the analytical gradient began with a flow rate of 0.33 μL/min. 0% MPB was held from 0 to 2 minutes, at 20 minutes MPB reached 16%, at 40 minutes MPB reached 72%, and at 42 minutes MPB reached 100%. The 100% MPB wash was held until 52 minutes and decreased back to 0% MPB at 54 minutes, equilibration of starting composition was held until 65 minutes. Source parameters used nitrogen as the drying gas with a temperature of 325°C flowing at 5L/min and a capillary voltage of 1800V. Auto MS/MS acquisition mode allowed 4 precursors per cycle with a threshold of 2000 counts and quadrupole filtering with a narrow isolation width for the 600–2000 m/z range. Collision-induced disassociation with N2 gas used a precursor-dependent formula y = 1.8*(m/z)/100 − 3.6 to determine individual collision energies with a range of 100–2000 m/z. Continual mass correction with a reference calibration standard of 1221.990637 m/z was used (Agilent Technologies, HP-1221). N-glycan compound verification used MassHunter Qual B08.00 software with the ‘Find by Molecular Feature’ algorithm and an in-house database containing N-glycan monoisotopic masses and descriptive information.

### Sphingolipid sample preparation

The membrane pellet from the initial steps was removed from − 20°C storage and allowed to acclimate to room temperature. Samples were resuspended in 500 μL of freshly prepared sphingolipid extraction solvent consisting of water/methanol/chloroform (3:8:4, v/v%) and sonicated for 15 minutes to assist in solvation. Proteins were precipitated by centrifugation at 8,800 RCF for 5 minutes and the supernatant was transferred to a new 1.5 mL Eppendorf vial. A bilayer phase separation was induced by the addition of 100 μL of 0.1 M KCl (MilliporeSigma, Cat#104936). The top aqueous-rich layer containing the sphingolipids of interest was transferred to a new vial and dried by vacuum centrifugation. Sphingolipid samples were cleaned up using a C-8 solid phase extraction plate (Glygen, Cat# FNSC08.800). SPE wells were first washed with elution solvent consisting of methanol/isopropyl alcohol (1:1, v/v%) and then conditioned in wash solvent consisting of methanol/water (1:1, v/v%). Samples were reconstituted with wash solvent, loaded onto the solid phase by gravity and the flow-through was reloaded. Sample wells were washed three times with 600 μL of washing solvent and all liquids were discarded. Sphingolipids were then eluted with 400 μL of elution solvent into a new collection plate. For each SPE step, the C-8 plate was allowed to gravity load to ensure analyte binding. Enriched sphingolipid samples were concentrated by drying to completion in vacuum centrifugation and reconstituted in 20 μL of water/methanol (1:1, v/v%) prior to analysis.

### Sphingolipid analysis methodology

Sphingolipid analysis was conducted with an Agilent 1200 series nanoflow HPLC equipped with a 10pt/2ps μ-valve(G1316-68709), nanoflow ESI operating in positive ion mode (G1992A), and a 6530 Q-TOF mass spectrometer. Chromatographic separations were carried out utilizing a Zorbax SB-300 C-8, 0.075 × 50 mm, 3.5 μm column (Agilent Technologies, Cat# 5065-9923). Four mobile phase compositions were used in this analysis with two low-flow pumps. The first pump (pump 1) operated at 0.7 μL/min and was used to load samples directly and wash the analytical column. The loading mobile phase (MPL) composition was 20 mM ammonium acetate in 56% methanol, 14% isopropyl alcohol, 0.1% glacial acetic acid and water (v/v). The washing mobile phase (MPW) composition was 50% methanol and 50% isopropyl alcohol (v/v). The second pump (pump 2) operated at 0.4 μL/min and was used to carry out the analytical gradient. Mobile phase A (MPA) had a composition of 20 mM ammonium acetate in 0.1% glacial acetic acid and water (v/v). Mobile phase B (MPB) had a composition of 20 mM ammonium acetate in 0.1% glacial acetic acid, 20% isopropyl alcohol, and 80% methanol (v/v). Samples were cooled with a 4°C sample tray and 1μL was injected and loaded directly onto the analytical column for 4 minutes with 100% MPL. At 4 minutes the μ-valve switched positions moving the analytical column into pump 2’s flow path and the gradient began. 70% MPB was held from 4 to 6 minutes, at 35 minutes MPB reached 93%, and at 36 minutes MPB reached 100%. The 100% MPB was held until 39 minutes and decreased back to 70% at 40 minutes, equilibration of the starting composition was held until 47 minutes. Source parameters used nitrogen as the drying gas with a temperature of 300°C flowing at 5 L/min and a capillary voltage of 1000V. Auto MS/MS acquisition mode allowed 5 precursors per cycle with a threshold of 600 counts and quadrupole filtering with a narrow isolation width for the 600–2000 m/z range. Collision-induced disassociation with N2 gas used a precursor-dependent formula y = 1.2* (m/z)/100 + 12 to determine individual collision energies with a range of 100–2000 m/z. Continual mass correction with a reference calibration standard of 1221.990637 m/z was used (Agilent Technologies, HP-1221). Sphingolipid compound verification used MassHunter Qual B08.00 software with the ‘Find by Molecular Feature’ algorithm and an in-house database containing sphingolipid monoisotopic masses and descriptive information.

### Statistical analysis

**Figure 1:**
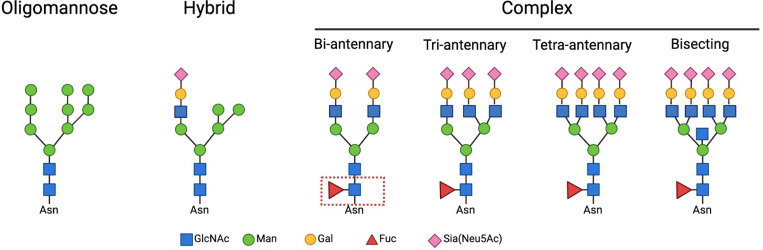
Outline of N-glycan classes. Examples from left to right represent oligomannose, hybrid, and complex N-glycans. Complex N-glycans can be classified as bi-, tri-, tetra-antennary, and bisecting N-glycans based on the number of starting GlcNAc residues on the mannose residues. The core fucosylation is marked with a red dash square. Recommended symbol nomenclature was used. GlcNAc: N-acetylglucosamine; Man: mannose; Gal: galactose; Fuc: fucose; Sia: sialic acid.

Single glycans with missing values for more than 80% of samples were excluded from further analysis (Supplementary Fig. 1). The fractions of glycan subtypes (as shown in Fig. 1), and sialylated and fucosylated N-glycans were calculated as the sum of relative abundances of single glycans. Differential abundance analysis was performed as linear regression models with the glycan abundance as the dependent variable and the treatment as the independent variable in R. ANOVA tests and contrasts between treatments and control were evaluated. The AβO and LPS groups were compared to the control group using Dunnett’s test. The differences of the three treatment groups on the PCA plot were evaluated using the MANOVA test followed by post-hoc linear discriminant analysis (LDA).

## RESULTS

### Both AβO and LPS induce significant changes in the N-glycan profiles of hiMG

**Figure 2:**
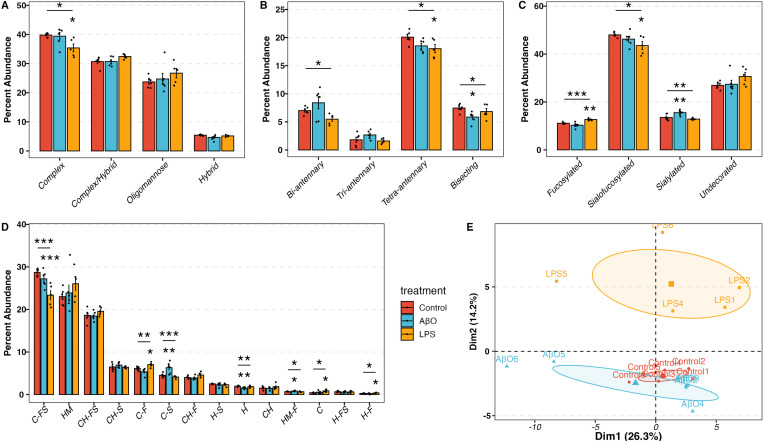
N-glycomic profile in control and AβO-, LPS-activated hiMG. A) the percent abundance of complex, complex/hybrid, oligomannose and hybrid N-glycan. B) The percent abundance of bi-, tri-, tetra-, and bisecting complex N-glycan. C) The percent abundance of each decoration, including fucosylated, sialofucosylated, sialylated, and undecorated N-glycan. D) The percent abundance of each decoration on each N-glycan type. Glycans are named as “glycan type-decoration”. C, CH, H, HM represent Complex, Complex/Hybrid, Hybrid, and Oligomannose, respectively. S, F, SF represent sialylated, fucosylated, and sialofucosylated, respectively. E) Principal Component Analysis (PCA) plot showing the clustering of control, AβO- and LPS-activated samples. MANOVA p-value =0.0014. Red, blue, and yellow correspond to control, AβO, and LPS groups. The asterisk (*) marks above the segments indicate the p-values obtained from the one-way ANOVA test. The asterisk marks below the segments indicated the p-values from the Dunnett test for the corresponding treatment group. p-values *<0.05, **<0.01, and ***<0.001.

We analyzed the N-glycan profiles of control (mock treatment), AβO-activated, and LPS-activated hiMG using LC-MS/MS technology, with six replicates per group. We treated hiMG for 24 hr with 3 μM AβO and 100 ng/ml LPS, optimal concentrations to induce pro-inflammatory cytokines IL-1β, TNF-α, and IL-6 ^22^. Figure 1 illustrates the N-linked glycosylation types. In all groups, the complex-type N-glycans were the most abundant N-glycans in hiMG, making up ~ 35–40% of total N-glycans (Fig. 2A). This is in keeping with the general composition of brain glycans ^29^. Hybrid glycans were the least abundant in hiMG, making up less than 10% of total N-glycans (Fig. 2A). Complex-type N-glycans can be further classified as bi-, tri, and tetra-antennary, as well as bisecting N-glycans, which contains an additional GlcNAc bound to the core (Fig. 1). In hiMG, the tetra-antennary structure constituted the predominant subtype of complex-type N-glycans, followed by bi-antennary, bisecting, and tri-antennary structures (Fig. 2B). N-Glycans can be decorated by adding fucose, sialic acid, or both to the terminal end of glycans. Fucose can also be added to the core structure. These modifications are important for protein functions such as protein stability, cell adhesion, signal transduction, immunological responses, and cell-to-cell interactions 30,31. We quantified such modifications and found that in hiMG, sialofucosylated N-glycans were the most abundant, making up ~ 50% of glycans. About 30% of N-glycans were not decorated by fucose or sialic acid residues. The rest of N-glycans were only decorated by either fucose or sialic acid residues (Fig. 2C). Considering both subtypes and decorations, sialofucosylated complex N-glycans and undecorated oligomannose N-glycans are the two most abundant structures in hiMG (C-FS and HM in Fig. 2D). AβO and LPS treatments had a significant impact on the N-glycan profile of hiMG as shown by the increased dispersion of the AβO-activated hiMG compared to the control cluster and the separation of the LPS-activated hiMG clusters from the control cluster on the PCA plot (MANOVA p-value = 0.0014). The distance between the LPS cluster and the control cluster was greater than the distance between the AβO cluster and the control cluster, indicating that LPS might induce a higher degree of glycosylation changes than AβO (Fig. 2E, Supplementary Fig. 2A).

### Decreased bisecting N-glycans in AβO-activated hiMG

**Figure 3:**
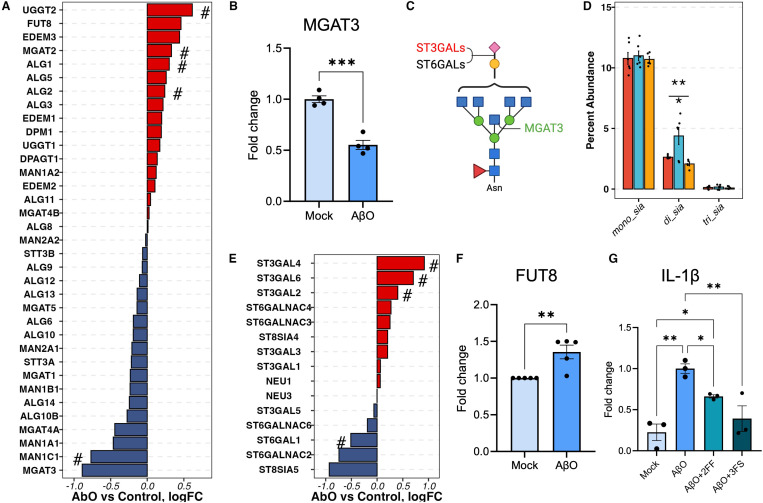
Sialylation changes in AβO-activated hiMG. A) The differential expression of genes involved in the biosynthesis of N-glycan core structure. B) q-PCR measurements of MGAT3. C) The schematics of the N-glycan structure and the corresponding enzymes. D) The percent abundance of mono-, di-, and tri- sialic acid residues among sialylated only N-glycans. E) The differential expression of genes involved in sialylation. F) q-PCR measurements of FUT8. G) Fucosylation and sialylation inhibitors reduce pro-inflammatory cytokine IL-1β production induced by AβO treatment, shown by q-PCR; N=3, Student-Newman-Keuls test. Red, blue, and yellow denote control, AβO, and LPS groups. For A) and E), the pound (#) denotes genes with unadjusted p-value < 0.05 but FDP >0.05. For D), red, blue, and yellow correspond to control, AβO, and LPS groups. The asterisk (*) marks above the segments indicate the p-values obtained from the one-way ANOVA test. The asterisk marks below the segments indicated the p-values from the Dunnett test for the corresponding treatment group. p-values *<0.05, **<0.01, and ***<0.001.

According to the N-glycome, AβO, but not LPS, treatment reduced the percent abundance of bisecting N-glycans in hiMG (Fig. 2B). Consistent with this glycomic change, differential expression (DE) analysis of RNA-seq data revealed that following AβO treatment, beta-1,4-mannosyl-glycoprotein 4-beta-N-acetylglucosaminyltransferase 3 (MGAT3) was the most downregulated gene across all genes involved in the core N-glycan formation process (Fig. 3A), which was further confirmed by q-PCR (Fig. 3B). MGAT3 encodes N-acetylglucosaminyltransferase-III (GnT-III), which is uniquely responsible for transferring a GlcNAc residue to the mannose of the trimannosyl core of N-glycans to produce a bisecting N-glycan ^32^ (Fig. 3C). Thus, the downregulation of the MGAT3 gene could be the transcriptional basis of the reduction of bisecting N-glycans observed in AβO treated hiMG cells.

### Increased sialylation contributed to AβO-induced pro-inflammatory response

AβO treatment also increased the percent abundance of sialylated N-glycans (Fig. 2C), in particular, sialylated complex N-glycans (C-S in Fig. 2D). We therefore further explored the changes in the number of sialic acid residues on N-glycans and found that the increased sialylation was largely attributed to an increased percent abundance of di-sialylated N-glycans (Fig. 3D). DE analysis of RNA-seq data showed that among the genes encoding sialyltransferases and sialidases, ST3GAL2, ST3GAL4, and ST3GAL6 were significantly upregulated by AβO treatment (Fig. 3E). Given that ST3GALs encode for enzymes responsible for transferring sialic acid to the galactose residues with α−2-3 linkage (Fig. 3C), the results suggest that α−2,3-linked sialic acids possibly contributed to the increased di-sialylation observed in the AβO-activated hiMG. Of note, ST3GAL4 and ST3GAL6 preferably catalyze the sialylation on N-glycans ^33^, while ST3GAL2 exclusively catalyzes the sialylation of gangliosides and mucin-type O-glycans ^34^.

DE analysis and q-PCR also revealed increased levels of FUT8 transcript encoding α1-6 fucosyltransferase (fucosyltransferase 8), which is uniquely responsible for core fucosylation, in AβO-treated hiMG (Fig. 3A, F). This result corroborates our published data reporting that AβO enhances FUT8-catalyzed core fucosylation, which is a signaling pathway required for AβO-induced microglia activation 22. Moreover, inhibition of core fucosylation using 2-fluoro-fucose (2FF) alleviated AβO-induced overexpression of pro-inflammatory cytokine IL-1β in hiMG with a significant reduction in IL-1β expression in cells co-treated with 2FF (Fig. 3G).

Additionally, to investigate the role of sialylation in AβO-induced hiMG activation, we inhibited sialylation by co-treating hiMG with 2,4,7,8,9-Penta-O-acetyl-N-acetyl-3-fluoro-β-D-neuraminic acid methyl ester (3FS). Similar to the effects observed upon fucosylation inhibition, sialylation inhibition significantly reduced AβO-induced pro-inflammatory response, resulting in IL-1β expression levels returning to those of mock-treated cells, with no significant differences detected (Fig. 3G).

### Decreased complex N-glycans in LPS-activated hiMG

**Figure 4:**
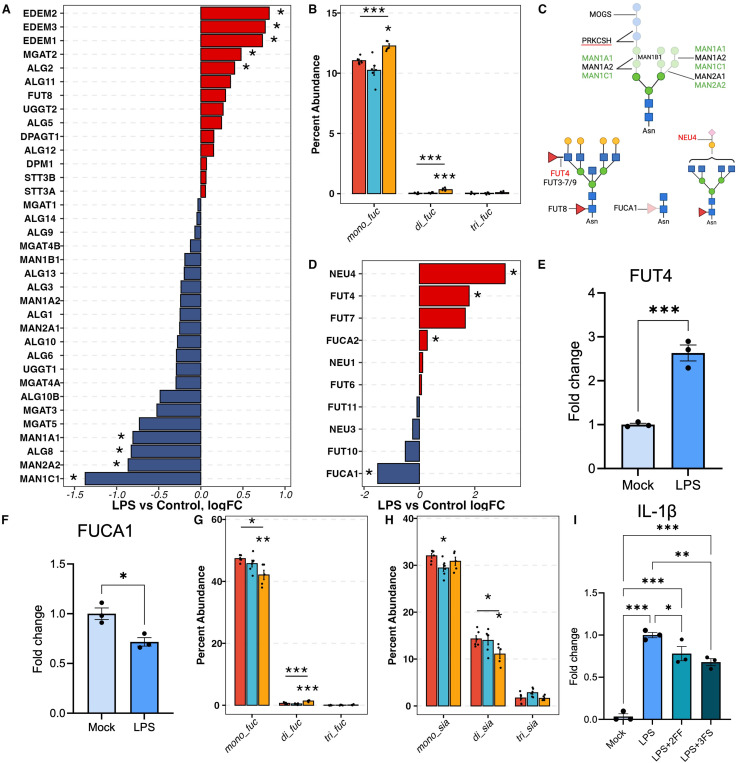
Fucosylation changes in LPS-activated hiMG. A) The differential expression of genes involved in the biosynthesis of N-glycan core structure and mannosidases. B) The percent abundance of mono-, di-, and tri-fucose residues among fucosylated only N-glycans. C) The schematics of complex N-glycan biosynthesis, fucosylation, and sialylation, including the enzymes involved. Red, blue, and yellow denote control, AβO, and LPS groups. D) The differential expression of genes involved in fucosylation and sialylation. E) and F) q-PCR measurements of FUT4 and FUCA1. G) The percent abundance of mono-, di-, and tri-fucose residues among sialofucosylated N-glycans. H) The percentage abundance of mono-, di-, and tri-sialic acid residues among sialofucosylated N-glycans. I) Fucosylation and sialylation inhibitors reduce pro-inflammatory cytokine IL-1β production induced by LPS treatment, shown by q-PCR; N=3, Student-Newman-Keuls test. For A) and D) The asterisk (*) denotes genes with FDP < 0.05. For B) G), and H), red, blue, and yellow correspond to control, AβO, and LPS groups. The asterisk (*) marks above the segments indicate the p-values obtained from the one-way ANOVA test. The asterisk marks below the segments indicated the p-values from the Dunnett test for the corresponding treatment group. p -values *<0.05, **<0.01, and ***<0.001.

In contrast to AβO-activated hiMG, LPS-activated hiMG exhibited a reduced percent abundance of complex-type N-glycans, specifically the tetra-antennary complex N-glycans (Fig. 2A, B). Complex N-glycans are derived from oligomannose N-glycans. In the endoplasmic reticulum (ER) and cis-Golgi, α-mannosidase I (MAN1B1), α1-2 mannosidases IA, IB, and IC (encoded by MAN1A1, MAN1A2, and MAN1C1, respectively) trim mannose residues from oligomannose N-glycans to yield Man5GlcNAc2, a key intermediate in the pathway to generate hybrid and complex N-glycans. Conversely, misfolded glycoproteins are degraded in the ER with the binding of ER degradation-enhancing α-mannosidase-like proteins (EDEM1-3) ^35^. As a result, these proteins are not processed into complex N-glycans nor transported to the membrane ^35,36^. In the DE analysis of hiMG transcriptomic data, MAN1A1, MAN1C1, and MAN2A2 were significantly downregulated and EDEM1-3 were significantly up-regulated in LPS-treated hiMG (Fig. 4A). The transcript levels of other key mannosidases, including MAN1B1, MAN1A2, and MAN2A1, were also decreased though the differences did not reach statistical significance (Fig. 4A). The downregulation of these mannosidases and upregulation of EDEMs may contribute to the reduced abundance of complex N-glycans.

### Increased fucosylation and decreased sialofucosylation associated with LPS-induced pro-inflammatory response

The changes in LPS-activated hiMG also differed from AβO-activated hiMG in terms of fucosylation and sialylation. The extent of fucosylation (i.e., fucosylated-only N-glycans), especially complex- and hybrid-type N-glycans, was increased in LPS-activated hiMG but remained unchanged in AβO-activated hiMG (Fig. 2C–D). This overall change was the sum of changes of individual glycan structures. Fucosylated-only N-glycans with one and two fucose residues increased in LPS-activated hiMG (Fig. 4B), which contributed to the overall increased percent abundance of fucosylated N-glycans.

Fucosyltransferases (FUTs) 3–7 and FUT9 are responsible for transferring fucose to antenna GlcNAc residues (Fig. 4C). According to the DE analysis and q-PCR, FUT4 was significantly upregulated in LPS-treated hiMG (Fig. 4D, E), which possibly contributed to increased fucosylation, especially increased di-fucosylated N-glycans. Fucose residues on N-glycans can be hydrolyzed by fucosidases (FUCAs). Alpha-L-Fucosidase 1 (FUCA1) specifically hydrolyzes the fucose residue at the core structure (Fig. 4C). The transcript level of FUCA1 was significantly downregulated as shown by RNA sequencing and q-PCR (Fig. 4D, F), suggesting less hydrolysis of core fucosylated N-glycans. This, together with enhanced FUT4 action, could lead to increased percent abundance of fucosylated N-glycans.

In contrast, sialofucosylated N-glycans, the most abundant types, were significantly reduced overall in LPS-activated hiMG, especially sialofucosylated complex N-glycans (Fig. 2C–D). The observed decrease in sialofucosylated N-glycans may be attributed to reduced activity of fucosyltransferases, sialyltransterases, or both. The DE analysis highlighted that neuraminidase 4 (NEU4) was overexpressed in LPS-activated hiMG, potentially contributing to the decreased abundance of sialofucosylated N-glycans (Fig. 4D). Interestingly, among the sialofucosylated N-glycans those containing one fucose residue decreased while those containing two fucose residues increased (Fig. 4G). Meanwhile, sialofucosylated N-glycans containing two sialic acid residues decreased, consistent with the overexpression of sialyltransferases (Fig. 4H). Thus, the decrease in sialofucosylated complex-type N-glycans was the primary effect of LPS treatment, specifically characterized by a decreased in mono-fucosylated and di-sialylated N-glycans and concomitant increase in di-fucosylated N-glycans.

To investigate the role of fucosylation and sialylation in LPS-activated hiMG, fucosylation and sialylation were inhibited using 2FF and 3FS, respectively. Both compounds significantly attenuated the LPS-induced overexpression of IL-1β (Fig. 4I).

### The glycosphingolipid (GSL) profile of hiMG

**Figure 5:**
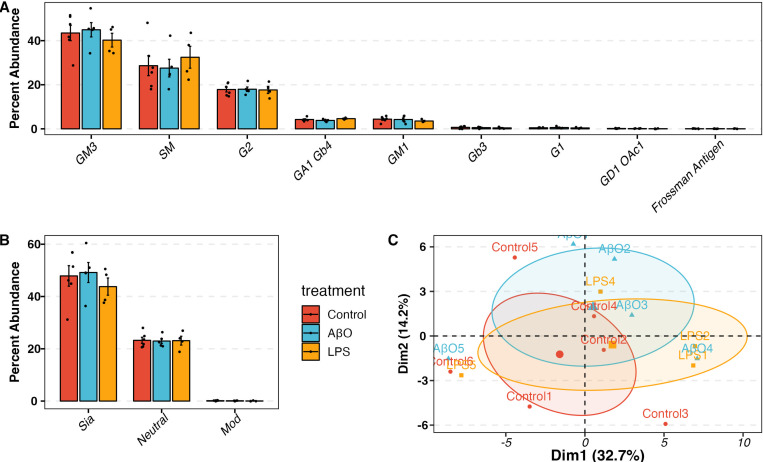
Glycosphingolipid (GSL) profile in control and AβO-, LPS-activated hiMG. A) The percent abundance of GSL subtypes. G1 and G2 refer to unknown GSL cores with 1 or 2 hexoses. B) The percent abundance of decorated GSLs. Mod refers to GSLs modified with O-acetylated. C) Principal Component Analysis (PCA) plot showing the clustering of control, AβO- and LPS-activated samples. MANOVA p-value =0.56. Red, blue, and yellow correspond to control, AβO, and LPS groups.

GSLs are a subclass of lipids composed of a ceramide part and a mono- or oligosaccharide part. In the adult mammalian brain, the major GSLs are the gangliosides of GM1, GD1a, GD1b, and GT1b that have one to three sialic acid residues respectively ^37,38^. Because sialic acid residues on a glycoprotein or glycolipid may modulate cell signaling mediated by lectins such as selectins and sialic acid-binding immunoglobulin-type lectins (Siglecs) and because we found significant changes in sialic acid levels following AβO and LPS treatment, we profiled the GSL composition as well. The most abundant GSL was ganglioside GM3, the precursor of other complex gangliosides (Fig. 5A). The second most abundant species was sphingomyelin (SM), a ceramide with a phosphocholine headgroup, followed by neutral GSL G2. The remaining GSLs were less abundant with each accounting for less than 5%. Regarding sialylation, GSLs detected in hiMG were predominantly sialylated GSLs (Fig. 5B). Interestingly, the GSL profile did not change significantly in response to either AβO or LPS activation. The PCA plot showed that the clusters of control, AβO, and LPS groups did not separate from each other (MANOVA p-value = 0.56, Fig. 5C, Supplementary Fig. 2B). The genes involved in GSL biosynthesis also did not show significant changes in response to AβO activation (Supplementary Fig. 3A). In LPS-activated hiMG, even though the genes involved in GSL biosynthesis, i.e., UGCG, B4GALT5, and B4GALNT1, were significantly upregulated, no significant changes were observed when individual GSLs in either treatment were examined (Fig. 5A, Supplementary Fig. 3B).

## DISCUSSION

The composition and balance between various types of glycans play an important role in regulating cell functions ^39^. Cellular N-glycans can be subclassified as complex-, oligomannose-, and hybrid-type N-glycans. Oligomannose N-glycans are the precursor of hybrid and complex-type N-glycans. Mannosidases hydrolyze mannose from the oligomannose structure, preparing it for complex- and hybrid-type N-glycan biosynthesis. Oligomannose glycans have been reported as the major N-glycan type in the undifferentiated human monocytic leukemia cell line THP-1, primary blood-derived CD14 + monocytes, and in Caco-2 cells ^40–43^. After differentiation, the levels of oligomannose glycans in these cells decreased, accompanied by an increase in complex-type structures. The latter became the predominant N-glycan type in differentiated cells. In line with the differentiated state of microglia, hiMG expresses predominantly complex-type N-glycans regardless of activation. On the other hand, our hiMG data (summarized below), when compared to published data from other cell types, show differences mainly in levels of glycan decorations such as mono- or di-sialylation, fucosylation, or sialofucosylation, suggesting the significance of these modifications in regulating microglia-specific functions.

**Figure 6:**
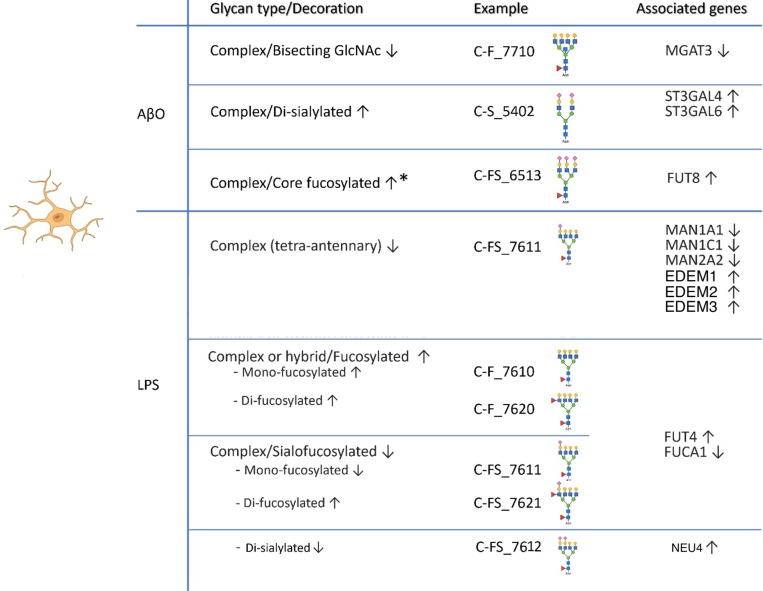
Summary of glycosylation and associated gene expression changes in hiMG stimulated by AβO and LPS. Representative examples of indicated glycan structures measured by mass spectrometry (MS) are listed in the middle column, and the genes linked to the indicated glycosylation changes identified by RNA-seq are listed in the right column. Note that current glycomic methods cannot precisely distinguish between core fucose and terminal fucose, and that the fucose residues in the structures such as C-FS_6513 can be either at the core or the terminal position. *The increased core fucosylation following AβO treatment is based on Jin et al., 2023.

The differential glycosylation changes stimulated by AβO and LPS are summarized in Fig. 6. Congruent evidence from both glycomic and transcriptomic analyses reveals the transcriptional basis of these changes and enhances the validity of our findings. We found that AβO induces increased sialylation catalyzed by ST3GALs and decreased bisecting N-glycans catalyzed by GnT-III. A third major glycosylation change, previously reported by us ^22^, is increased core fucosylation catalyzed by FUT8, which is corroborated in the current study by increased expression of FUT8 transcript. These three glycosylation changes are known to affect immune regulation ^30,39,44^, suggesting their significance in microglial function. The inhibition of core fucosylation and sialylation in our study significantly attenuated the expression of pro-inflammatory cytokine IL-1β in hiMG induced by AβO treatment. The observed glycosylation changes in AβO-activated hiMG have also been implicated in AD, as published data using brain or cerebrospinal fluid samples from participants with AD or mild cognitive impairment (MCI) highlighted altered levels of bisecting N-glycans, sialylation, and core fucose in N-glycans ^9,45–48^. Altered glycoforms of individual AD-relevant proteins were also reported; for example, the familial AD mutant of the Aβ-amyloid precursor protein (APP) was shown to have a higher content of bisecting GlcNAc and core fucose residues compared to wild-type APP ^49^. However, exact glycosylation-regulated molecular mechanisms, especially those pertaining to neuroinflammation in AD are largely unknown. Furthermore, a widely recognized feature of glycobiology is that different cell types express distinct glycosyltransferase isozymes, and thus proteins may have different glycosylation patterns depending on their cell of origin and in response to functional needs ^50^. Therefore, analyses of multicellular brain tissues or other cell types do not precisely reflect glycosylation alterations in microglia. In this context, the microglia- and stimulant-specific glycosylation changes we report here would provide the first significant insight into how glycosylation alterations may regulate microglial function.

Among the three specific glycosylation changes, sialylation is the most studied in AD due to the high level of interest in Siglec, in part based on the association of the gene of CD33, a Siglec receptor, to AD risk ^51^. Neuronal membranes contain a high density of sialic acid residues on glycoproteins, which protect neurons from aberrant microglial phagocytosis^52^ via interacting with Siglec receptors such as CD22 and CD33 on microglia ^53–55^. However, how sialylation changes in microglia *per se* affect neuron-microglia interaction or microglial function is poorly understood. Previous studies using the mouse BV2 microglia cell line found that BV2 cells activated by LPS, fibrillar Aβ, and tau had increased sialidase activity that desialylated the BV2 cell surface, promoting phagocytosis of neurons mediated by CR3 ^53,56^. Although we found that oligomeric Aβ-activated hiMG showed an increase in total sialylation, the RNA-seq analysis suggests that the changes in sialylation may be specific to glycosidic linkages, with a decrease in α2-6 and α2-8 linked sialic acids and an increase in α2-3 linked sialic acids. In regard to CD33 ligand binding preference, evidence points to overlapping specificities for α2-3 and α2-6 linked sialic acids^57^ where the binding affinity was increased with sulfation^58,59^ and decreased with branched α1-3 fucosylation ^60^.Of note, AβO treatment of hiMG was accompanied by reduced phagocytosis of pHrodo Green BioParticles and of Aβ itself as demonstrated previously ^22^, revealing the complexity and need for further investigation. The differences may be explained by different cell models (BV2 vs. hiMG), interspecies differences (mouse vs. human), different modes of microglia activation (fibrillary Aβ or LPS vs AβO), and different methods of analysis. We used human iPSC-derived microglia as the model due to its strong human relevance, while the BV2 model is, unlike authentic microglia, a rapidly growing tumor cell line with well recognized limitations ^61^. AβO is considered a primary pathological agent in early stages of AD preceding Aβ fibril formation ^62^, and the respective influences on microglia state by AβO and Aβ fibril may represent different neuroinflammation phases in AD.

Regarding increased core fucosylation, our recent study using the same hiMG model provides the first indication about its significance in microglia activation ^22^. We further found that FUT8 expression was increased in both human AD brains and microglia isolated from 5xFAD mice, a model of AD-like cerebral amyloidosis. Moreover, FUT8 is a component of the p53 signaling cascade regulating microglial behavior; FUT8-catalyzed core fucosylation is required for AβO-activated microglial alterations, including induction of pro-inflammatory cytokines, activation of p38MAPK, and phagocytic deficits ^22^.

Bisecting GlcNAc, a β1,4-linked GlcNAc attached to the core β-mannose residue, is a structure specific to complex N-glycans. It is a prominent feature of human brain complex N-glycans, occurring at a frequency of 40% ^63^. The presence of a bisecting GlcNAc on glycoproteins has many implications in biological functions, such as in immune tolerance ^64^, tumor metastasis, and brain development ^65,66^. The relevance of bisecting N-glycans to AD is suggested by its altered levels in AD brain samples together with altered expression of GnT-III mRNA. However, whether it is up- or down-regulated in AD remains inconclusive as conflicting results were reported ^45–47^. Interestingly, APP and β-site APP cleaving enzyme-1- (BACE1), two key proteins required for Aβ production, contain glycosylation modifications with bisecting N-glycans. It was reported that Aβ (aggregation state not characterized) treatment enhanced GnT-III mRNA expression in Neuro2a mouse neuroblastoma cells. This was considered neuroprotective as GnT-III-transfected cells showed increased α-secretase activity and decreased production of Aβ40 and Aβ42 ^46^. Apparently contradicting this notion, Kizuka et al. reported that modifications with bisecting N-glycans stabilized BACE1 to increase Aβ production and that GnT-III deficiency reduced Aβ-plaque formation in the brain by accelerating lysosomal degradation of BACE1 ^67^. Again, the significance of altered bisecting N-glycans modifications in microglia is poorly understood. Our observation of decreased GnT-III mRNA expression and decreased bisecting N-glycans abundance in AβO-activated hiMG provides the first indication that bisecting N-glycans modifications play a role in regulating microglial function.

Compared to AβO, LPS clearly induced a distinct glycosylation-related transcriptomic and glycomic pattern with decreased abundance of complex N-glycans and increased abundance of fucosylation, consistent with their different signaling pathways. The combination of increased FUT4 and decreased FUCA1 expression may underlie the increased overall fucosylation we found in LPS-activated hiMG. However, in contrast to AβO effects, we did not find increased core fucosylation following LPS treatment 22, suggesting that LPS mainly enhances terminal fucosylation. In addition, the fucosylation changes were differential among glycan structure subgroups with the overall increase in fucosylation consisting predominantly of increased fucosylated-only N-glycans, while the decrease in sialofucosylation specifically characterized by a reduction in mono-fucosylated and di-sialylated N-glycans. Though the glycosylation changes observed in this study contrast with those in LPS-treated HMC cells in terms of oligomannose, complex, and sialofucosylated N-glycans ^68^, our functional measurements with sialylation and fucosylation inhibition support the role of these glycan modifications in the pro-inflammatory response. The inhibition of sialylation and fucosylation both reduced the expression of the pro- inflammatory cytokine IL-1β in LPS-activated hiMG. However, unlike in AβO-activated hiMG, neither inhibitor restored IL-1β expression to comparable levels in mock-treated cells. This may be due to 2FF primarily inhibiting core fucosylation, whereas LPS treatment predominantly enhances terminal fucosylation. Furthermore, LPS treatment did not significantly increase sialylation in hiMG as AβO treatment, potentially explaining why inhibiting sialylation had a weaker effect on the pro-inflammatory response in LPS-treated cells compared to AβO-treated cells. More functional implications of these novel structural changes warrant further investigation. Our results also prompt a novel hypothesis that LPS downregulates mannosidases in the N-glycan biosynthesis pathway, which trim mannose residues from oligomannose N-glycans to yield Man5GlcNAc2, a key intermediate in the pathway to generate hybrid and complex N-glycans. Meanwhile, LPS upregulates mannosidases in the ER-associated protein degradation pathway, which trim mannose residues from oligomannose N-glycans on misfolded proteins, accelerating their degradation. Together, these processes could result in reduced levels of complex N-glycans. This hypothesis would need further investigation.

Little is known about GSL in microglia, despite reported changes in ganglioside profiles in neurodegenerative disorders ^69^ and the anti-inflammatory effects of GM1 and other gangliosides on microglia ^70^. It was observed by dot blot analysis that microglia were characterized by abundant GM1 in mixed murine glia culture, and that treatment of mouse primary microglia with LPS resulted in decreased GM1 and GT1b levels ^70,71^. However, these previously reported changes were not apparent in our GSL profiling of hiMG. We identified no GSL alterations in hiMG following AβO or LPS stimulation. The most abundant GSL was ganglioside GM3, the precursor of other complex gangliosides (Fig. 4A). The second most abundant sphingolipid was SM followed by ganglioside G2. The remaining GSLs were less abundant with each accounting for less than 5%. When considering the number of sialic acids, GSLs detected in hiMG were predominantly sialylated GSLs (Fig. 5B).

In summary, using a human microglia culture model, we were able to identify differential AβO- and LPS-induced glycosylation changes that may impact the functional or cellular interaction outcomes of these two types of pro-inflammatory activation, and we did not identify significant GSL changes. Our data add to currently scant information about microglia- and stimulant-specific “glycosylation codes”. They also help generate hypotheses that may lead to a better glycoproteomic and glycolipidomic understanding of microglial function, which is exponentially more complex than the glycomic landscape we report here.

## Supplementary Material

Supplement 1

## Figures and Tables

**Table 1.** Primers and primer sequences used for qPCR assays

Gene (Invitrogen)	Primer Sequence
IL-1β (Human)	FW: GTGCAGTTCAGTGATCGTACAGG RV: CCACAGACCTTCCAGGAGAATG
β-Actin (Human)	FW: TCAAGATCATTGCTCCTCCTGAG RV: ACATCTGCTGGAAGGTGGACA

Gene (Bio-Rad)	Primer
FUT4 (Human)	qHsaCED0048069
FUT8 (Human)	qHsaCID0008117
FUCA1 (Human)	qHsaCID0017362
MGAT3 (Human)	qHsaCED00820404

## Data Availability

The RNA sequencing data has been deposited to the Gene Expression Omnibus (GSE277259). N-glycomic and glycosphingolipid quantification data are provided in the supporting information. The raw data are available from Carlito B. Lebrilla upon request after acceptance.
