# Supplementary material for: Unique N-glycosylation signatures in Aβ oligomer-and lipopolysaccharide-activated human iPSC-derived microglia
Source: Res Sq. 2024 Nov 19:rs.3.rs-5308977. Preprint. [Version 1] doi: 10.21203/rs.3.rs-5308977/v1 (PMC11601871; doi:10.21203/rs.3.rs-5308977/v1)
Supplement: Supplement 1 [file NIHPPRS5308977V1-supplement-1.pdf]

Supplementary Files

This is a list of supplementary files associated with this preprint. Click to download.

Supportinginformation.docx

iPSCrawdatagslabsoluteabundances.csv
iPSCrawdatanglycanrelativeabundances.csv
